# “Similia Similibus Curentur”: The scientific grounding of the homeopathic therapeutic principle through the systematic study of the rebound effect of modern drugs

**DOI:** 10.1016/j.clinsp.2022.100091

**Published:** 2022-07-31

**Authors:** Marcus Zulian Teixeira

**Affiliations:** Departamento de Psiquiatria, Faculdade de Medicina, Universidade de São Paulo (USP), São Paulo, SP, Brazil

## Abstract

• Homeopathy employs the principle of therapeutic similitude as a method of treatment.
• Primary action of drugs is followed by secondary and opposite reaction of the organism.
• This secondary and opposite reaction of the organism is namely the rebound effect of drugs.
• Homeopathy employs the rebound effect of drugs in a therapeutic way.

## Introduction

Homeopathy has been a Brazilian medical specialty since 1980 and is based on four epistemological premises with several research lines attesting its scientific validity^1^^,^^2^: principle of therapeutic similitude (principle of cure by similars), experimentation of medicines in healthy individuals (homeopathic pathogenetic trials), use of individualized medicines (in accordance with the characteristic symptomatic totality) and dynamized doses (doses diluted and agitated serially). Among these premises, the principle of therapeutic similitude *(similia similibus curentur)* is the main scientific foundation and was described by several exponents of medicine since Hippocrates in ancient Greece.

## Principle of therapeutic similitude

In developing the homeopathic treatment method, Samuel Hahnemann (1755‒1843) grounded the principle of similitude in attentive observations of the effects of contemporary drugs on human health. In employing the phenomenological method of qualitative research, he described the occurrence of a “biphasic action” on dozens of palliative drugs of his time: after the “direct primary action of the drug” was observed, the consequent and opposite “indirect secondary action of the body” then followed (*Organon of medicine,* paragraphs 59 and 65).^3^ The authors quote a detailed description of the biphasic actions of “opium” by Hahnemann as an example.

“Important symptoms of persistent diseases have never yet been treated with such palliative, antagonistic remedies, without the opposite state, a relapse ‒ indeed, a palpable aggravation of the malady ‒ occurring a few hours afterward. […] for frequent waking at night the physician prescribed in the evening, without heeding the other symptoms of the disease, opium, which by virtue of its primary action produced the same night (stupefied, dull) sleep, but the subsequent nights were still more sleepless than before; ‒ to chronic diarrheas, he opposed, without regarding the other morbid signs, the same opium, whose primary action is to constipate the bowels, and after a transient stoppage of diarrhea it subsequently became all the worse; ‒ violent and frequently recurring pains of all kinds he could suppress with opium for but a short time; they then always returned in greater, often intolerable severity, or some much worse affection came in their stead. For the longstanding nocturnal cough, the regular physician knew no better than to administer opium, whose primary action is to suppress every irritation; the cough would then perhaps cease the first night, but during the subsequent nights it would be still more severe, and if it were again and again suppressed by this palliative in increased doses, fever and nocturnal perspiration were added to the disease; […]”. (*Organon of medicine,* paragraph 59).^3^

Justifying its biphasic action of antagonistic (antipathic or palliative) drugs on the automatic manifestation of “our life-preserving power” (“homeostasis”, according to modern physiology), Hahnemann suggests a physiological explanation to the principle of similitude (primary action of the drug followed by secondary and opposite action of the body): “Every agent that acts upon the vitality, every medicine, deranges more or less the vital force, and causes a certain alteration in the health of the individual for a longer or a shorter period. This is termed *primary action*. […] To its action our vital force endeavors to oppose its own energy. This resistant action is a property, is indeed an automatic action of our life-preserving power, which goes by the name of *secondary action* or *counteraction*” (*Organon of medicine,* paragraph 63).^3^

Adding hundreds of examples of accidental homeopathic cures described in the literature (*Organon of medicine*, Introduction)^3^ to his empirical observations, and employing the inductive Aristotelian reasoning *(modus ponens)*, Hahnemann enunciates the principle of therapeutic similitude (principle of cure by similars): any medicine which causes certain signs and symptoms in their primary action in healthy individuals can be used, in their secondary action, to cure similar signs and symptoms in sick individuals *(similia similibus curentur)* (*Organon of medicine*, paragraphs 24‒27).^3^

Contrariwise, demonstrating the occurrence of evident aggravation of diseases after ceasing the palliative effect of antipathic or palliative treatments (principle of cure by contraries) (*Organon of medicine*, paragraphs 57‒61),^3^ Hahnemann reinforces the validity of homeopathic treatment according to the deductive Aristotelian reasoning (*modus tollens*) or “indirect proof”.

“Important symptoms of persistent diseases have never yet been treated with such palliative, antagonistic remedies, without the opposite state, a relapse ‒ indeed, a palpable aggravation of the malady ‒ occurring a few hours afterward. For a persistent tendency to sleepiness during the day the physician prescribed coffee, whose primary action is to enliven; and when it had exhausted its action the day ‒ somnolence increased; ‒ for frequent waking at night he gave in the evening, without heeding the other symptoms of the disease, opium, which by virtue of its primary action produced the same night (stupefied, dull) sleep, but the subsequent nights were still more sleepless than before; ‒ to chronic diarrheas, he opposed, without regarding the other morbid signs, the same opium, whose primary action is to constipate the bowels, and after a transient stoppage of diarrhea it subsequently became all the worse; ‒ violent and frequently recurring pains of all kinds he could suppress with opium for but a short time; they then always returned in greater, often intolerable severity, or some much worse affection came in their stead. […] weakness of the bladder, with consequent retention of urine, was sought to be conquered by the antipathic work of cantharides to stimulate the urinary passages whereby evacuation of the urine was certainly at first effected but thereafter the bladder becomes less capable of stimulation and less able to contract, and paralysis of the bladder is imminent; ‒ with large doses of purgative drugs and laxative salts, which excite the bowels to frequent evacuation, it was sought to remove a chronic tendency to constipation, but in the secondary action the bowels became still more confined; […] severely burnt parts feel instantaneous alleviation from the application of cold water, but the burning pain afterwards increases to an incredible degree, and the inflammation spreads and rises to a still greater height […] How often, in one word, the disease is aggravated, or something even worse is effected by the secondary action of such antagonistic (antipathic) remedies, the old school with its false theories does not perceive, but experience teaches it in a terrible manner.” (*Organon of medicine*, paragraph 59).^3^

Therefore, the homeopathic treatment method employs the secondary action of the body in a therapeutic way by administering the medicines which cause similar symptoms in healthy individuals to ill individuals in order to awaken a healing reaction of the body against their own diseases. By emphasizing that such secondary action of the body (vital reaction) is observed “in each and every instance with no exceptions”, with ponderable or infinitesimal doses and in both healthy and ill individuals, Hahnemann raised the principle of cure by similars to the level of a “natural law of cure” (*Organon of medicine*, paragraphs 58, 61, 110‒112).^3^

“Had physicians been capable of reflecting on the sad results of the antagonistic employment of medicines, they had long since discovered the grand truth, *that the true radical healing art must be found in the exact opposite of such an antipathic treatment of the symptoms of disease*; they would have become convinced, that as a medicinal action antagonistic to the symptoms of the disease (an antipathically employed medicine) is followed by only transient relief, and after that is passed, by invariable aggravation, the converse of that procedure, the homeopathic employment of medicines according to the similarity of symptoms, must affect a permanent and perfect cure, if at the same time the opposite of their large doses, the most minute doses, are exhibited. […]” (*Organon of medicine,* paragraph 61).^3^

## Scientific grounding of the homeopathic therapeutic principle in modern pharmacology

Bridging the gap between homeopathic pharmacology and conventional, the “primary action” described by Hahnemann corresponds to the “therapeutic, adverse and side effects” of conventional drugs, while the “secondary action” corresponds to the “rebound effect” or “paradoxical reaction” of the body, observed after discontinuation of numerous classes of modern drugs which act contrary (antagonist, antipathic or palliative) to the signs and symptoms of diseases.

By pharmacological concepts, the rebound effect is the emergence or re-emergence of symptoms that were either absent or controlled while taking a drug; that is, if a drug produces a rebound effect, the condition is used to treat may come back even stronger when the drug is withdrawn, discontinued or loses effectiveness (tolerance or tachyphylaxis).

The rebound effect causes stronger or more frequent symptoms than the ones originally suppressed, which arise at different intervals and last for varying periods. Usually, the rebound phenomenon manifests itself after finishing the therapeutic effect of the drug (biological half-life).

Since 1998, following the deductive Aristotelian reasoning employed by Hahnemann to validate the principle of therapeutic similitude, the authors have been scientifically grounding the homeopathic therapeutic principle through the systematic study of the rebound effect of several classes of modern drugs,^4^, ^5^, ^6^, ^7^, ^8^, ^9^, ^10^, ^11^, ^12^, ^13^, ^14^, ^15^ demonstrating the occurrence of a strong secondary and opposite reaction of the body after ceasing the primary action of the palliative or antagonist drugs (Table 1 and Table 2).

**Table 1:** Primary action (therapeutic effect) of modern drugs followed by secondary action and opposite (rebound effect or paradoxical reaction) of the body.

Primary action (therapeutic effect) of modern drugs	Secondary action (rebound effect or paradoxical reaction) of the body
Hypotension action (α−2 agonists, beta-blockers, ACE inhibitors, MAO inhibitors, nitrates, sodium nitroprusside and hydralazine)	Paradoxical arterial hypertension
Antithrombotic action (argatroban, bezafibrate, heparin, salicylates, warfarin and clopidogrel)	Rebound thromboembolism
Antianginal action (nitrates, beta-blockers and calcium channel blockers)	Paradoxical increase of frequency and/or intensity of angina pectoris
Antiarrhythmic action (adenosine, amiodarone, β-blockers, calcium channel blockers, disopyramide, flecainide, lidocaine, mexiletine, moricizine and procainamide)	Rebound exacerbation of basal arrhythmia after discontinuation or withdrawal of drug
Pleiotropic (vasoprotective) action (statins)	Paradoxical endothelial dysfunction
Anxiolytic action (barbiturates, benzodiazepines and carbamates)	Rebound anxiety
Sedative-hypnotic action (barbiturates, benzodiazepines, morphine, promethazine and zopiclone)	Paradoxical increased of agitation, nervousness, restlessness and irritability
Antidepressant action (tricyclic, MAO inhibitors and selective serotonin reuptake inhibitors,	Rebound increase of depressive symptoms
Antipsychotic action (clozapine, phenothiazines, haloperidol and pimozide)	Paradoxical exacerbation of psychotic manifestations

**Table 2:** Primary action (therapeutic effect) of modern drugs followed by secondary action and opposite (rebound effect or paradoxical reaction) of the body.

Primary action (therapeutic effect) of modern drugs	Secondary action (rebound effect or paradoxical reaction) of the body
Antidyspeptic action (antacids, H_2_ antagonists, misoprostol, sucralfate, and protons pump inhibitors)	Paradoxical increase in the production of hydrochloric acid and gastrin
Bronchodilator action (short- and long-acting beta-adrenergic agonists, sodium cromoglycate, ipratropium and nedocromil,	Rebound bronchoconstriction
Analgesic action (caffeine, calcium channels blockers, clonidine, ergotamine, methysergide, opiates and salicylates)	Paradoxical hyperalgesia after discontinuation or withdrawal of drug
Anti-inflammatory action (steroids, ibuprofen, indomethacin, paracetamol, and salicylates)	Rebound increase of inflammation
Diuretic action (furosemide, torasemide and triamterene)	Paradoxical retention of sodium and potassium with consequent increase of blood volume and arterial pressure
Antiresorptive action (bisphosphonates, denosumab and odanacatib)	Rebound increase of osteoclastic activity causing paradoxical atypical fractures
Immunomodulatory action (glucocorticoids, interferon, recombinant monoclonal antibodies, and tumor necrosis factor inhibitors)	Paradoxical effects on the inflammatory and immune response of drugs

These scientific pieces of evidence^4^, ^5^, ^6^, ^7^, ^8^, ^9^, ^10^, ^11^, ^12^, ^13^, ^14^, ^15^ demonstrate that the properties of the rebound effect of modern pharmacology are similar to the secondary action of homeopathic pharmacology (*Organon of medicine,* paragraphs 59, 64, 69)^3^: 1) It induces a body reaction opposed to and of greater intensity compared to the primary action of drugs; 2) It takes place after the end of the primary action of the drug, and as the automatic manifestation of the body; 3) It does not depend on the type of drug, dose, treatment duration or category of symptoms (disease); 4) Its magnitude is proportional to the intensity of the primary action of the drug; and 5) It appears only in susceptible individuals (idiosyncrasy).

In accordance with its idiosyncratic nature, the rebound phenomenon appears in a small proportion of susceptible individuals; nevertheless, scientific pieces of evidence point to the occurrence of severe and fatal adverse events as a result of the paradoxical reaction of the body following the discontinuation of different categories of drugs.^4^, ^5^, ^6^, ^7^, ^8^, ^9^, ^10^, ^11^, ^12^, ^13^, ^14^, ^15^ This corroborates the magnitude of the phenomenon, the need to be duly known by health care providers, and the benefits of its therapeutic application according to the similitude principle.

## New homeopathic medicines: use of modern drugs according to the principle of similitude

Since 2003, assuming Hahnemann's postulate that the principle of therapeutic similitude is a “natural law of cure”, the authors have been suggesting the homeopathic use of modern drugs employing the rebound effect in a therapeutic way,^16^, ^17^, ^18^, ^19^, ^20^, ^21^, ^22^ proposing to prescribe drugs, in dynamized doses, which present a similar set of signs and symptoms in their primary action to the manifestations of sick individuals.

Entitled “New Homeopathic Medicines: use of modern drugs according to the principle of similitude”, this proposal encompasses 1250 modern drugs and has been available since 2021 in three free-access digital books indexed in the Virtual Health Library^23^: ”Scientific basis of the principle of similitude in modern pharmacology”^24^; “Homeopathic materia medica of modern drugs”^25^; and “Homeopathic repertory of modern drugs”.^26^

Thus, the authors developed a clinical research protocol^27^ to use potentized estrogen (17-ß estradiol) in treating endometriosis-associated pelvic pain in order to test this proposal, since estrogen causes, as adverse events, a similar set of signs and symptoms to endometriosis syndrome. This randomized clinical trial showed significant improvement compared to placebo in relation to pain, depression, and quality of life,^28^ thereby suggesting the scientific validity of this proposal.

## Discussion

Upon describing the undesirable effects of indiscriminate use of drugs according to the contrary principle, Hahnemann called attention to the risks derived from their secondary action (rebound effect or paradoxical reaction), resulting in “more serious disease or frequently even danger to life and death itself”. In turn, he validated the therapeutic similitude principle through the Aristotelian *modus tollens*: “If these ill-effects are produced, as may very naturally be expected from the antipathic employment of medicines, the ordinary physician imagines he can get over the difficulty by giving, at each renewed aggravation, a stronger dose of the remedy, whereby an equally transient suppression is effected; and as there then is a still greater necessity for giving ever-increasing quantities of the palliative there ensues either another more serious disease or frequently even danger to life and death itself, but never a cure of a disease of considerable or of longstanding.” (*Organon of medicine,* paragraph 60).^3^

Bridging between the therapeutic similarity principle and modern scientific reason, hundreds of studies in the medical literature describe the occurrence of secondary reactions following and opposed to the primary actions of many categories of drugs, thus corroborating the homeopathic assumption.^4^, ^5^, ^6^, ^7^, ^8^, ^9^, ^10^, ^11^, ^12^, ^13^, ^14^, ^15^ Such secondary action or reaction, which occurs automatically and instinctively to maintain the system homeostasis, is described by contemporary pharmacology and physiology as a rebound effect of drugs or a paradoxical reaction of the body, respectively. Analogously, the primary action of the drugs mentioned by Hahnemann represents the therapeutic, adverse, and side effects of modern drugs.

By definition, the intensity and/or frequency of the rebound effect are higher compared to the original symptoms, suppressed by the primary action of the drug. This characteristic distinguished the rebound effect from the natural return of chronic symptoms after the end of treatment. While drug discontinuation is a requisite for the occurrence of a rebound effect, it might also appear during treatment, as a function of the development of tolerance or therapeutic failure.

In conventional therapeutics, a large number of iatrogenic events might be avoided where health care providers pay attention to the possible occurrence of a rebound effect,^14^ and worsening of diseases is minimized by tapering. While not conventionally described or included the effects of discontinuation of drugs are a part of the pharmacology of any drug and thus ought to be considered in the teaching of modern pharmacology.

By employing the rebound effect of conventional drugs with curative intent the authors might broaden the scope of therapeutic similitude through the addition of hundreds of “new homeopathic medicines”. Such new homeopathic medicines cover signs and symptoms absent in the classical homeopathic pathogenetic trials and will allow treating countless modern disorders, diseases, and syndromes with homeopathy.^16^, ^17^, ^18^, ^19^, ^20^, ^21^, ^22^

## Conclusion

The wide dissemination of these two decades of studies in this research line will enable researchers and physicians to use them to scientifically validate the therapeutic similitude principle and to develop new homeopathic treatments for various health disorders and diseases.

## Conflicts of interest

The authors declare no conflicts of interest.
